# Enhancement of *O*-GlcNAcylation on Mitochondrial Proteins with 2-(4-Methoxyphenyl)ethyl-2-acetamido-2-deoxy-β-d-pyranoside, Contributes to the Mitochondrial Network, Cellular Bioenergetics and Stress Response in Neuronal Cells under Ischemic-like Conditions

**DOI:** 10.3390/molecules26195883

**Published:** 2021-09-28

**Authors:** Hui Xu, Mingzhi Du, Yuntian Shen, Yumin Yang, Fei Ding, Shu Yu

**Affiliations:** Key Laboratory of Neuroregeneration of Jiangsu and Ministry of Education, Co-Innovation Center of Neuroregeneration, NMPA Key Laboratory for Research and Evaluation of Tissue Engineering Technology Products, Nantong University, 19 Qixiu Road, Nantong 226001, China; 13862964186@163.com (H.X.); 13853204312@163.com (M.D.); syt517@ntu.edu.cn (Y.S.); yangym@ntu.edu.cn (Y.Y.)

**Keywords:** 2-(4-methoxyphenyl)ethyl-2-acetamido-2-deoxy-b-d-pyranoside, cellular bioenergetics, mitochondrial homeostasis, *O*-GlcNAcylation, oxygen glucose deprivation/reoxygenation stress, neuroprotection

## Abstract

*O*-GlcNAcylation is a nutrient-driven post-translational modification known as a metabolic sensor that links metabolism to cellular function. Recent evidences indicate that the activation of *O*-GlcNAc pathway is a potential pro-survival pathway and that acute enhancement of this response is conducive to the survival of cells and tissues. 2-(4-Methoxyphenyl)ethyl-2-acetamido-2-deoxy-β-d-pyranoside (SalA-4g), is a salidroside analogue synthesized in our laboratory by chemical structure-modification, with a phenyl ring containing a para-methoxy group and a sugar ring consisting of *N*-acetylglucosamine. We have previously shown that SalA-4g elevates levels of protein *O*-GlcNAc and improves neuronal tolerance to ischemia. However, the specific target of SalA-4g regulating *O*-GlcNAcylation remains unknown. To address these questions, in this study, we have focused on mitochondrial network homeostasis mediated by *O*-GlcNAcylation in SalA-4g’s neuroprotection in primary cortical neurons under ischemic-like conditions. *O*-GlcNAc-modified mitochondria induced by SalA-4g demonstrated stronger neuroprotection under oxygen glucose deprivation and reoxygenation stress, including the improvement of mitochondrial homeostasis and bioenergy, and inhibition of mitochondrial apoptosis pathway. Blocking mitochondrial protein *O*-GlcNAcylation with OSMI-1 disrupted mitochondrial network homeostasis and antagonized the protective effects of SalA-4g. Collectively, these data demonstrate that mitochondrial homeostasis mediated by mitochondrial protein *O*-GlcNAcylation is critically involved in SalA-4g neuroprotection.

## 1. Introduction

*O*-GlcNAcylation is a dynamic and reversible post-translational modification process, which occurs on serine or threonine residues of numerous nucleocytoplasmic proteins, including transcription factors, cytoskeletal proteins, and kinases [1]. The addition and removal of *O*-linked-β-N-acetylglucosamine (*O*-GlcNAc) are catalyzed by *O*-GlcNAc transferase (OGT) and *O*-GlcNAcase (OGA), respectively. OGT attaches *O*-GlcNAc moieties to specific substrate proteins using UDP-GlcNAc as the only sugar donor, which is synthesized from glucose through the hexosamine biosynthetic pathway (HBP). HBP is a sensor of cellular nutritional status. The recycling of *O*-GlcNAcylation rapidly occurs in response to cellular metabolic changes through the HBP [2,3,4].

*O*-GlcNAcylation is remarkably ubiquitous in the mammalian brain and acts as a metabolic sensor that links glucose metabolism to normal neuronal function [5,6]. Its dysregulation is involved in the pathogenesis of neurological disorders, including ischemic stroke. When challenged by ischemia and reperfusion (I/R) stress, the activation of *O*-GlcNAcylation is hampered in post-ischemic brains, especially in aged animals. Importantly, pharmacological elevation of brain *O*-GlcNAcylation is neuroprotective in young and aged brains after ischemic stroke [7,8]. Therefore, these data convincingly suggest that activation of *O*-GlcNAcylation is a potential pro-survival pathway in ischemic stress, and that this pathway could be a promising target for therapeutic intervention following brain ischemia.

**Scheme 1:**
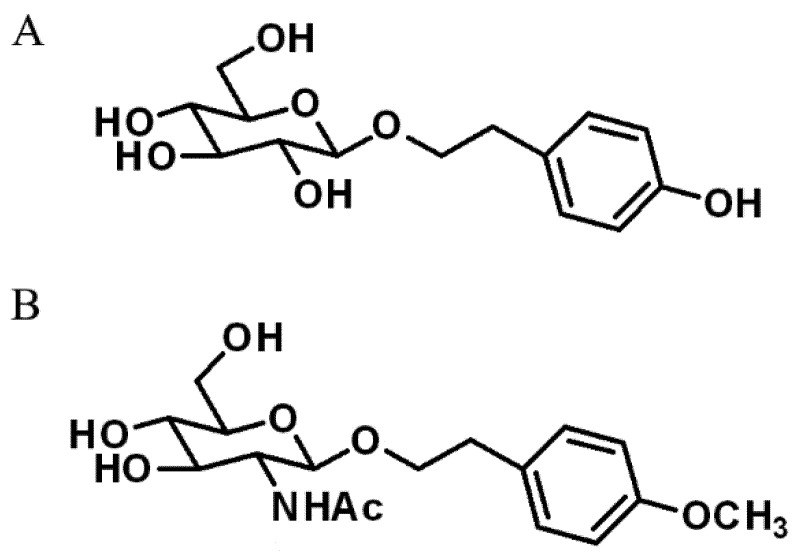
The chemical structure of salidroside (**A**) and SalA-4g (**B**).

Salidroside, a tyrosine-derived phenolic compound (Scheme 1A) with strong antioxidant activity, is a natural product extracted from medicinal plants under the Rhodiola genus [9]. Our early studies have found that salidroside exerts protective effects in vitro on nerve cells under various neurotoxic stress [10,11]. However, poor blood brain barrier (BBB) permeability and limited extraction efficiency may hinder the application of salidroside in the central nervous system (CNS). In order to improve BBB permeability of salidroside, we focused on the structural modification of salidroside for its better bioactivity in CNS. 2-(4-Methoxyphenyl)Ethyl-2-Acetamido-2-Deoxy-β-d-Pyranoside, a structural analogue of salidroside (code-named SalA-4g, Scheme 1B) synthetized by our laboratory, exhibits good BBB penetration ability, and exerts stronger neuroprotection in experimental ischemic stroke, as compared with salidroside [12,13]. Mechanistic studies indicate that SalA-4g improves neuronal energy metabolism under ischemia stress, and activation of *O*-GlcNAcylation plays pivotal roles in the pharmacological activity of SalA-4g [14,15,16]. However, the specific target of SalA-4g regulating *O*-GlcNAcylation is not clear.

Mitochondria, as the powerhouse of cells, are essential for maintaining homeostasis for energy-supplying in CNS [17]. Mitochondria form a dynamic and interconnected network, which plays an important role in cell energetics through the synthesis of adenosine triphosphate (ATP), maintenance of redox balance and calcium homeostasis, and regulation of cell death process [18,19]. Mitochondrial proteins are the main executors of mitochondrial function, which are finely regulated by multiple post-translational modifications. Notably, *O*-GlcNAcylation, as a nutrient sensor for metabolic status to cellular function, is involved in the regulation of mitochondrial metabolism, respiration, and dynamics [20,21]. To identify the exact contribution of SalA-4g to energy homeostasis of ischemic neurons, here, we hypothesize that mitochondrial bioenergy mediated by *O*-GlcNAcylation may be a key target for SalA-4g’s neuroprotection, and thereby clarify this important aspect.

## 2. Results

### 2.1. SalA-4g Reduces Cellular Injury and Enhances Cellular Bioenergy in Neuronal Cells under Oxygen-Glucose Deprivation/Reperfusion (OGD/R) Stress

**Figure 1:**
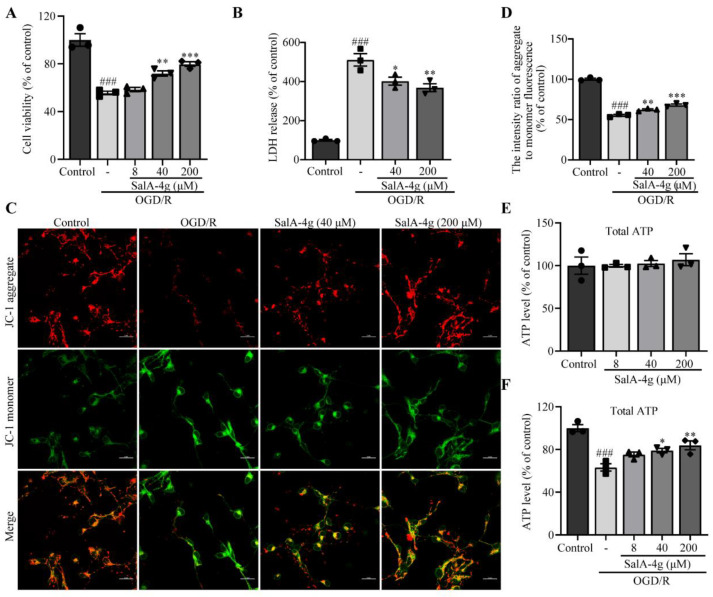
SalA-4g reduces cellular injury and enhances cellular bioenergy in cortical neurons under OGD/R stress. (**A**) Histogram depicting the cell viability of control cells, cells exposed to 2.5 h of OGD and 24 h of reoxygenation alone, and cells treated with different concentrations of SalA-4g during OGD/R period. (**B**) Histogram depicting the LDH release in neurons after treatments, as mentioned above. (**C**) Representative immunofluorescence of JC-1 staining. Scale bar, 25 μm. (**D**) Quantitative analysis of the intensity ratio of JC-1 aggregate to JC-1 monomer fluorescence in different groups. (**E**) Histogram depicting the total ATP levels in neurons exposed to different concentrations of SalA-4g for 24 h. (**F**) Histogram depicting the total ATP levels in neurons after treatments, as mentioned in A. All values are presented as mean ± SEM (*n* = 3); ^###^ *p* < 0.001 versus control; * *p* < 0.05, ** *p* < 0.01, and *** *p* < 0.001 versus OGD/R alone.

To evaluate the potential protective roles of SalA-4g on neuronal tolerance to OGD/R stress, we measured neuronal death and apoptosis by cell viability, lactate dehydrogenase (LDH) release, and mitochondrial membrane potential (MMP). The results showed that OGD/R markedly reduced the cell viability, and neurons treated with SalA-4g were resistant to the impairment, especially in the middle and high-dose groups (40 and 200 μM; Figure 1A). As expected, the release of LDH induced by OGD/R was also largely counteracted by SalA-4g (Figure 1B). Notably, the fluorescent staining and quantitative analyses of JC-1 showed that SalA-4g potently increased the ratio of J-aggregates (red) to monomers (green) in the OGD/R group, suggesting the recovery of MMP (Figure 1C,D). These data clearly indicate that SalA-4g possesses a strong neuroprotective ability against OGD/R insult in cortical neurons.

The disorder of energy metabolism after hypoxic/ischemic stress is the initiating factor of neuronal death [22]. Hence, we next examined the effect of SalA-4g on ATP levels of cortical neurons under normal and OGD/R stress conditions. SalA-4g had no significant effect on ATP levels of neurons under normal condition (Figure 1E). Importantly, OGD/R injury triggered an obvious reduction of cellular ATP production, which was reversed by SalA-4g treatment (Figure 1F), suggesting a beneficial effect of SalA-4g on maintaining cellular bioenergetics in neurons under OGD/R stress.

### 2.2. SalA-4g Enhances Mitochondrial Bioenergy and Stabilizes Mitochondrial Structure in Neuronal Cells under OGD/R Stress

**Figure 2:**
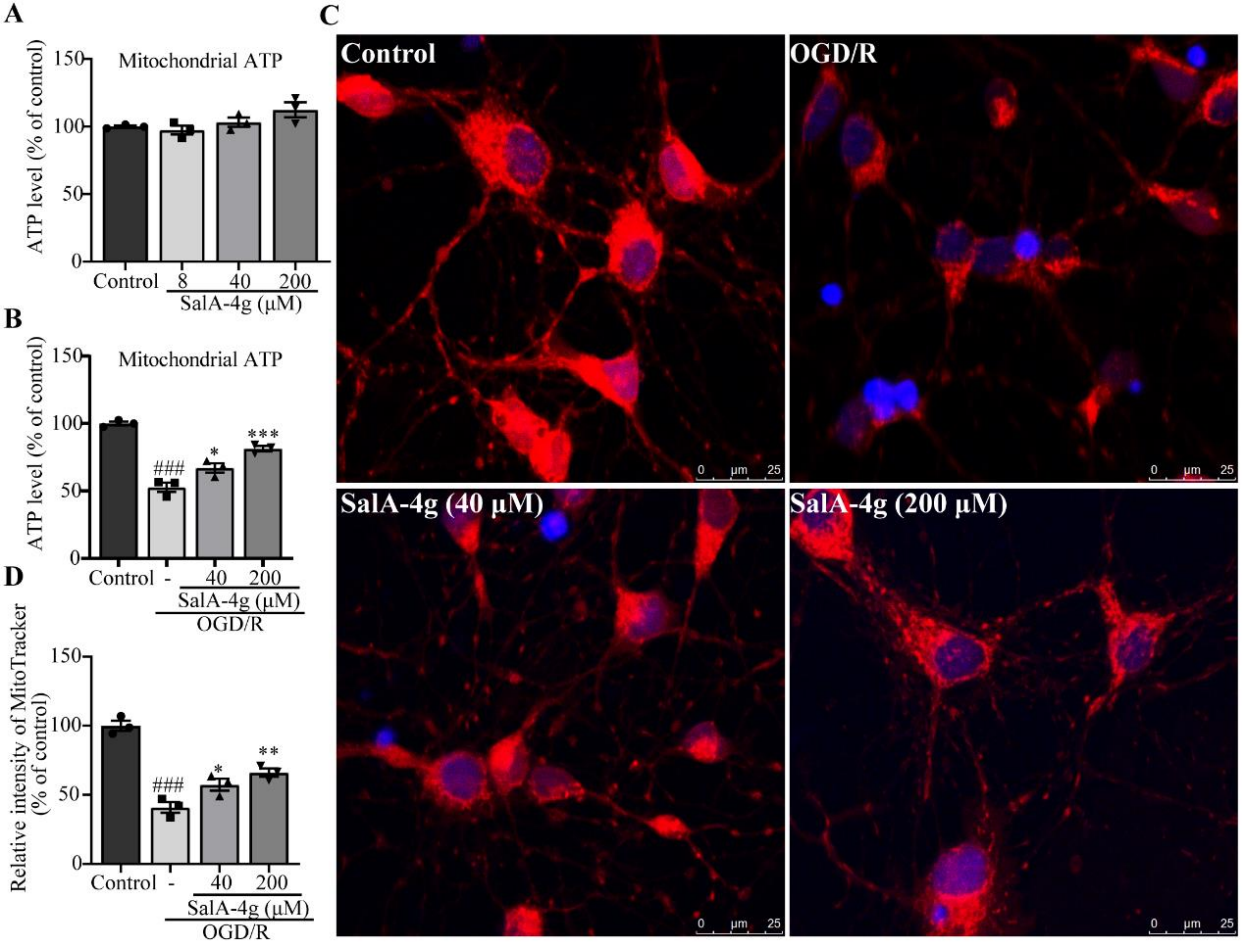
SalA-4g enhances mitochondrial bioenergy and activity in cortical neurons under OGD/R stress. (**A**) Histogram depicting the mitochondrial ATP levels in neurons exposed to different concentrations of SalA-4g for 24 h. (**B**) Histogram depicting the mitochondrial ATP levels of control cells, cells exposed to 2.5 h of OGD and 24 h of reoxygenation alone, and cells treated with different concentrations of SalA-4g during the OGD/R period. (**C**) Representative immunofluorescence of Mito-Tracker staining. Scale bar, 25 μm. (**D**) Histogram depicting the relative fluorescence intensity of Mito-Tracker in different groups. All values are presented as mean ± SEM (*n* = 3); ^###^ *p* < 0.001 versus control; * *p* < 0.05, ** *p* < 0.01, *** *p* < 0.001 versus OGD/R alone.

**Figure 3:**
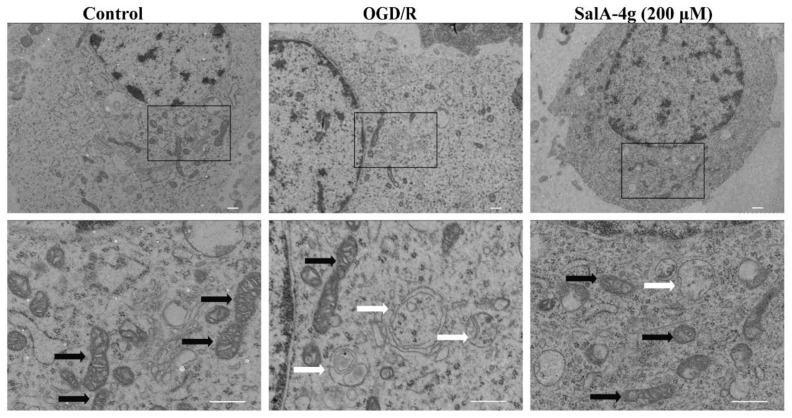
SalA-4g stabilizes the mitochondrial structure in cortical neurons under OGD/R stress. Representative TEM images of control cells, cells exposed to 2.5 h of OGD and 24 h of reoxygenation alone, and cells treated with 200 μM SalA-4g during OGD/R period. White arrows indicate damaged mitochondria and black arrows indicate healthy mitochondria. Scale bar, 0.5 μm.

Neurons are highly dependent on mitochondrial energy supply. Hypoxic/ischemic insult reduces mitochondrial oxidative phosphorylation and ATP production, leading to energy metabolism disorders and cell dysfunction [23]. Therefore, we hypothesized that mitochondria are the key targets of SalA-4g in improving the bioenergy homeostasis of neurons stressed by OGD/R. To test this hypothesis, a mitochondrial fraction was isolated and mitochondrial ATP production was further examined. As expected, SalA-4g had no effect on mitochondrial ATP in normal cultured neurons, but significantly antagonized the decrease of mitochondrial ATP level in neurons after OGD/R injury (Figure 2A,B). Moreover, the activity and distribution of mitochondria were examined by using a red fluorescent dye MitoTracker in living neurons. Morphological observation of MitoTracker staining showed pronounced dispersion of mitochondrial network and loss of active mitochondria in neurons induced by OGD/R insult, suggesting the disruption and depolarization of mitochondria (Figure 2C). Notably, SalA-4g effectively prevented these noticeable alterations in mitochondrial distribution and activity (Figure 2D). The ultrastructure of mitochondria was further examined by transmission electron microscopy (TEM). Normal mitochondria exhibited short rod-shaped or round structures with deep staining, as well as clearly identified membrane and cristae composition. OGD/R stress resulted in the destruction of mitochondrial structures, including the swelling and vacuolation of mitochondria, and the disappearance of cristae structure. Importantly, these severe changes of mitochondrial structure in injured neurons were partially prevented by SalA-4g treatment (Figure 3). Collectively, these results demonstrate that SalA-4g plays pivotal roles in maintaining mitochondrial structure and energy homeostasis in neuronal cells under OGD/R stress.

### 2.3. SalA-4g Alleviates Mitochondrial Oxidative Stress and Calcium Overload in Neuronal Cells under OGD/R Stress

**Figure 4:**
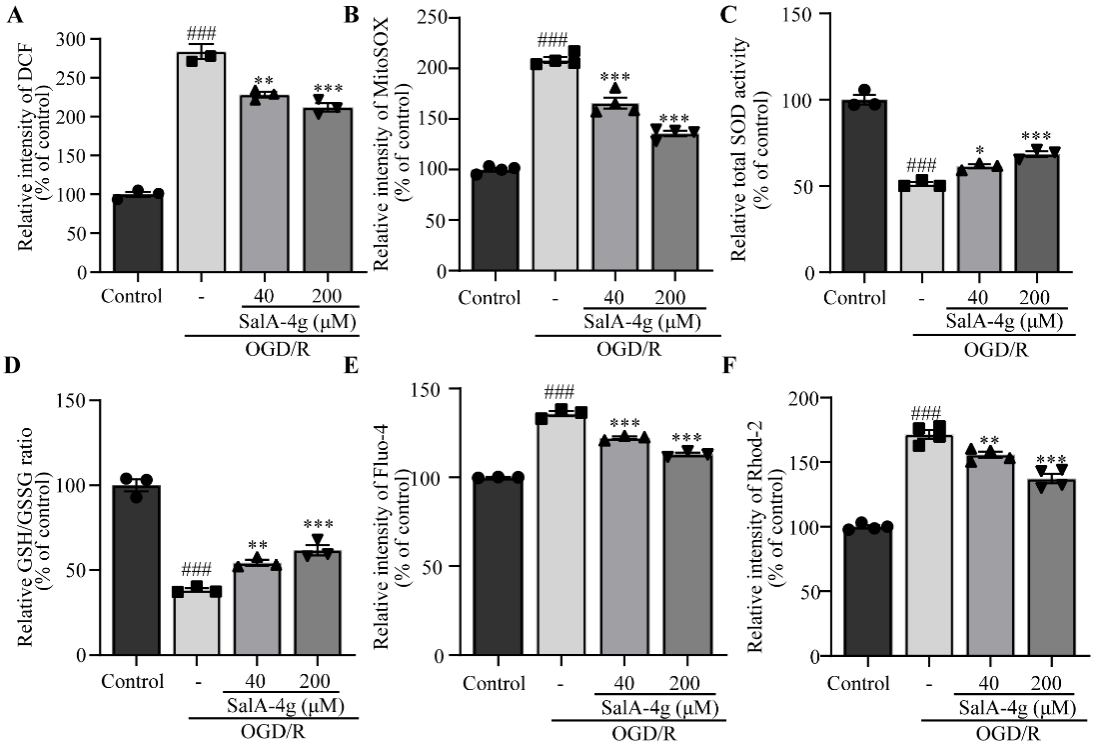
SalA-4g alleviates mitochondrial oxidative stress and calcium overload in cortical neurons under OGD/R stress. (**A**) The levels of intracellular ROS of control cells, cells exposed to 2.5 h of OGD and 24 h of reoxygenation alone, and cells treated with different concentrations of SalA-4g during OGD/R period. (**B**) The levels of mitochondrial superoxide in neurons after treatments, as mentioned above. (**C**) The total SOD activity in neurons after treatments, as mentioned above. (**D**) The ratio of reduced GSH to oxidized GSH (GSSG) in different groups. (**E**) Quantification of Fluo-4 fluorescence intensity in different groups. (**F**) Quantification of Rhod-2 fluorescence intensity in different groups. All values are presented as mean ± SEM (*n* = 3 or 4); ^###^ *p* < 0.001 versus control; * *p* < 0.05, ** *p* < 0.01, and *** *p* < 0.001 versus OGD/R alone.

Oxidative stress, a well-recognized pathological factor for cerebral ischemia, occurs as a result of the imbalance between oxidation and antioxidation [24]. Mitochondria are not only the main source of intracellular reactive oxygen species (ROS), but also an attack target of cellular redox stress. Stress induces the mitochondrial respiratory chain to generate excessive ROS, which destroys the mitochondrial respiratory chain and membrane structure, resulting in mitochondrial Ca^2+^ overload and excessive ROS generation, and finally forms a vicious cycle of oxidative stress [25]. We therefore examined the regulation of SalA-4g in oxidative stress and calcium imbalance in neurons under OGD/R stress. OGD/R induced a remarkable increase in both intracellular ROS and mitochondrial superoxide, while SalA-4g significantly diminished the excessive production of ROS and mitochondrial superoxide (Figure 4A,B). Moreover, SalA-4g augmented the activity of superoxide dismutase (SOD) and the ratio of reduced glutathione (GSH) to oxidized glutathione (GSSG), suggesting that an oxidation-reduction imbalance was ameliorated in OGD/R neurons treated by SalA-4g (Figure 4C,D). We also found an increase in both cellular and mitochondrial calcium accumulation in neurons induced by OGD/R, as detected by the Fluo-4 AM and Rhod-2 AM. Strikingly, these responses were drastically reduced in SalA-4g groups, with a dose-dependent manner (Figure 4E,F).

### 2.4. SalA-4g Inhibits Mitochondrial Apoptosis Pathway in Neuronal Cells under OGD/R Stress

**Figure 5:**
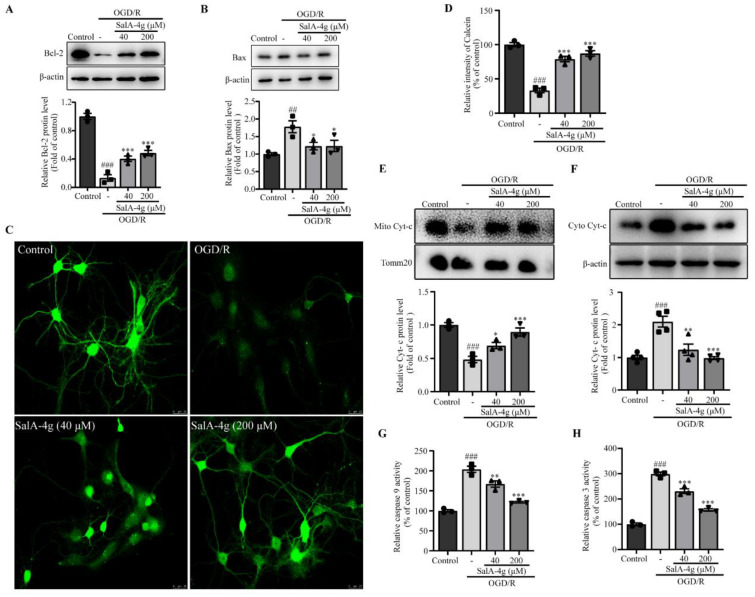
SalA-4g inhibits mitochondrial apoptosis pathway in cortical neurons under OGD/R stress. (**A**,**B**) Western blot analysis of Bcl-2 (**A**) and Bax (**B**) expression of control cells, cells exposed to 2.5 h of OGD and 24 h of reoxygenation alone, and cells treated with different concentrations of SalA-4g during OGD/R period. (**C**,**D**) Representative fluorescence images (**C**) and quantification of average fluorescence intensity (**D**) of calcein in different groups. Scale bar, 25 μm. (**E**,**F**) Western blot analysis of cytochrome c expression in mitochondria (**E**) and cytosol (**F**) in different groups. (**G**,**H**) Histogram depicting the activation of caspase 9 (**G**) and caspase 3 (**H**) in different groups. All values are presented as mean ± SEM (*n* = 3 or 4); ^##^ *p* < 0.01, ^###^ *p* < 0.001 versus control; * *p* < 0.05, ** *p* < 0.01, *** *p* < 0.001 versus OGD/R alone.

Beyond its well-recognized role in cellular bioenergetics, mitochondria are critical mediators of signals involved in various cellular functions, including apoptosis [26]. During apoptosis, an imbalance among the Bcl-2 family, calcium surplus and ROS induce mitochondrial permeability transition pore (MPTP) opening, which results in the release of mitochondrial contents, including cytochrome c, and finally activates the caspase-cascade system [27]. We therefore characterized these key mediators in mitochondrial apoptosis pathway in OGD/R-injured neurons. As shown in Figure 5A,B, Bcl-2 expression was downregulated while Bax was upregulated in neurons induced by OGD/R. However, this phenomenon was significantly antagonized after treatment with SalA-4g. We further evaluated OGD/R-induced MPTP opening as detected by calcein-AM. A remarkably reduced fluorescence intensity of calcein was seen in the OGD/R group, indicating the opening of a large number of MPTP. Notably, SalA-4g drastically blunted OGD/R-induced MPTP opening (Figure 5C,D). The expression of cytochrome c was further assessed in mitochondria and cytoplasm, respectively. SalA-4g significantly increased levels of cytochrome c in the mitochondria, whereas there was a corresponding decrease in cytoplasmic cytochrome c, implying the decline in release of cytochrome c from mitochondria (Figure 5E,F). Finally, SalA-4g successfully antagonized the activation of caspase 9 and caspase 3 in neuronal cells under OGD/R stress (Figure 5G,H). Collectively, these results suggest that SalA-4g exerts neuroprotective effects by inhibiting mitochondrial apoptosis pathway.

### 2.5. SalA-4g Reinforces O-GlcNAc Modification on Global and Mitochondrial Proteins in Neuronal Cells under Normal and OGD/R Conditions

**Figure 6:**
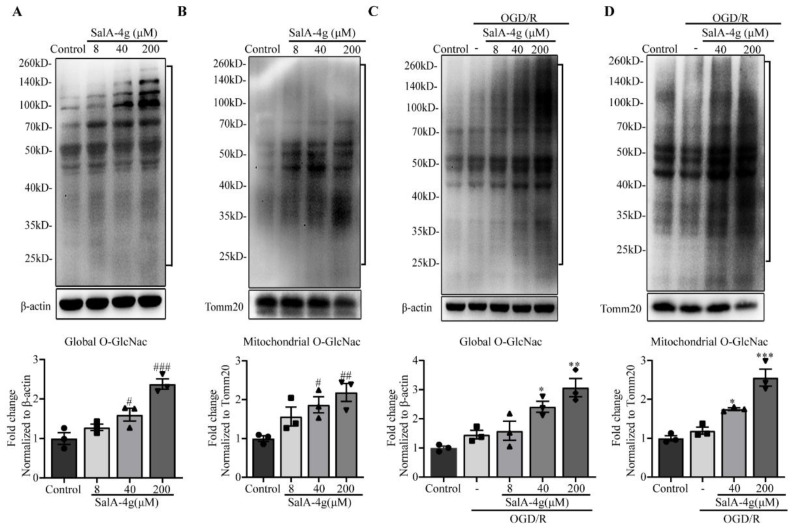
SalA-4g reinforces O-GlcNAc modification on global and mitochondrial proteins in cortical neurons under normal and OGD/R conditions. Global O-GlcNac levels (**A**) and mitochondrial O-GlcNac levels (**B**) in primary cortical neurons exposed to different concentrations of SalA-4g for 24 h. Global O-GlcNac levels (**C**) and mitochondrial O-GlcNac levels (**D**) of control cells, cells exposed to 2.5 h of OGD and 24 h of reoxygenation alone, and cells treated with different concentrations of SalA-4g during OGD/R period. Regions between 25 kDa and 260 kDa marked by brackets were used to quantify levels of O-GlcNAcylation. All values are presented as mean ± SEM (*n* = 3); ^#^ *p* < 0.05, ^##^ *p* < 0.01, and ^###^ *p* < 0.001 versus control; * *p* < 0.05, ** *p* < 0.01, and *** *p* < 0.001 versus OGD/R alone.

Next, we determined the effect of SalA-4g on the global *O*-GlcNAc level of cultured cortical neurons and found that the overall *O*-GlcNAcylation levels increased in a dose-dependent manner after SalA-4g treatment, peaking at 200 μM (Figure 6A). To characterize the role of SalA-4g on mitochondrial *O*-GlcNAc modification, mitochondria were isolated and *O*-GlcNAcylation on mitochondrial proteins was further identified. Similar results were obtained in mitochondrial *O*-GlcNAcylation levels treated with SalA-4g, with significant increases achieved by medium (40 μM) and high doses (200 μM) (Figure 6B).

We further confirmed the activation of *O*-GlcNAcylation by SalA-4g in cortical neurons in response to OGD/R stress. OGD/R induced a slight increase in the level of overall *O*-GlcNAcylation with no statistical significance. Importantly, a marked increase in *O*-GlcNAcylation with SalA-4g treatment was observed in a dosage-dependent manner (Figure 6C). We also found no significant change in protein *O*-GlcNAcylation on mitochondria in neurons under OGD/R stress, and, notably, there was a drastic increase in *O*-GlcNAc modification on mitochondria, especially in 40 μM and 200 μM SalA-4g groups (Figure 6D). Taken together, SalA-4g triggers the formation of *O*-GlcNAc in neurons under normal and OGD/R conditions.

### 2.6. O-GlcNAcylation on Mitochondrial Proteins Is Involved in SalA-4g Regulation of Mitochondrial Network Homeostasis in Neuronal Cells under OGD/R Stress

**Figure 7:**
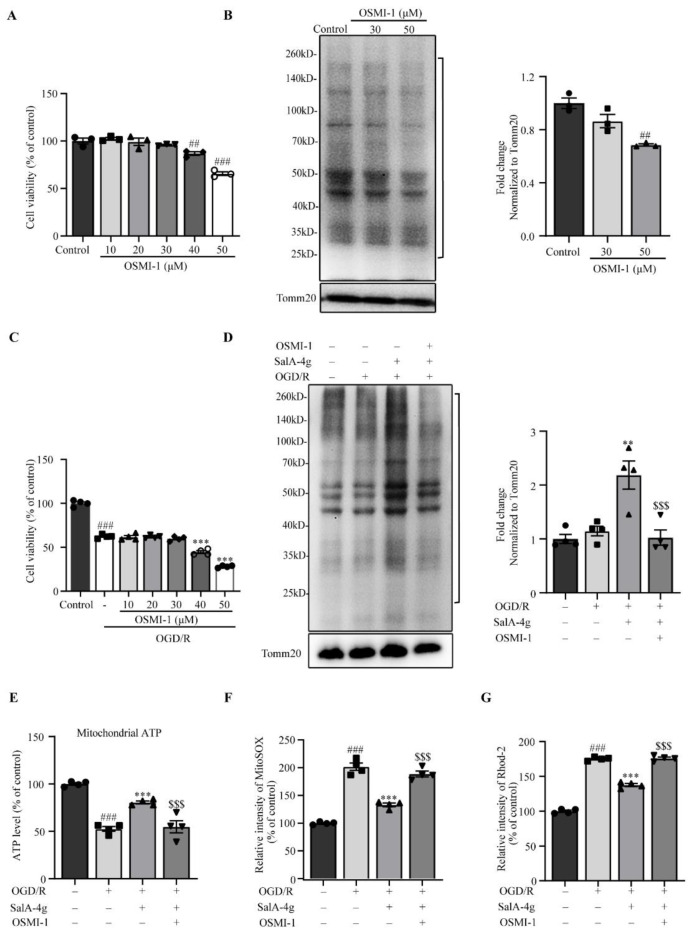
O-GlcNAcylation on mitochondrial proteins is involved in the regulation of SalA-4g on mitochondrial stress response in cortical neurons under OGD/R stress. (**A**) Histogram depicting the cell viability of primary cortical neurons exposed to different concentrations of OSMI-1 for 24 h. (**B**) Western blot analysis of mitochondrial O-GlcNAcylation in neurons exposed to 30 μM and 50 μM of OSMI-1 for 24 h. (**C**) Histogram depicting the cell viability of control cells, cells exposed to 2.5 h of OGD and 24 h of reoxygenation alone, and cells treated with different concentrations of OSMI-1 during the OGD/R period. (**D**) Western blot analysis of mitochondrial O-GlcNAcylation in OGD/R injury neurons with treatment of SalA-4g (200 μM) or SalA-4g (200 μM) + OSMI-1 (30 μM). Regions between 25 kDa and 260 kDa marked by brackets were used to quantify levels of O-GlcNAcylation. (**E**) Histogram depicting the mitochondrial ATP levels in different groups. (**F**) The levels of mitochondrial superoxide in different groups. (**G**) Quantification of the fluorescence intensity of Rhod-2 in different groups. All values are expressed as mean ± SEM (*n* = 4); ^##^ *p* < 0.01, ^###^ *p* < 0.001 versus control; ** *p* < 0.01, and *** *p* < 0.001 versus OGD/R alone; ^$$$^ *p* < 0.001 versus OGD/R + SalA-4g group.

To decipher a unique role of O-GlcNAc-modified mitochondria in SalA-4g regulation of mitochondrial stress under OGD/R injury, we used an OGT inhibitor OSMI-1 to antagonize the enhancement of *O*-GlcNAc modification induced by SalA-4g. First, cell viability assay was assessed to evaluate the toxic effect of OSMI-1 on neurons under normal conditions. OSMI-1 has shown no obvious neurotoxicity up to 30 μM, although there was a dose-dependent decrease in cell viability (Figure 7A). We further evaluated the effects of different doses of OSMI-1 on mitochondrial *O*-GlcNAc levels in normal neurons. We found that 30 μM OSMI-1 induced a slight decrease in mitochondrial *O*-GlcNAcylation level, while 50 μM OSMI-1 resulted in a significant decline in *O*-GlcNAcylation (Figure 7B). Next, the effect of OSMI-1 on neuronal viability under OGD/R conditions was examined. As shown in Figure 7C, the effects of different doses of OSMI-1 on the viability of OGD/R injured neurons were similar to those of normal neurons, indicating that 30 μM OSMI-1 is safe. Therefore, we selected 30 μM OSMI-1 for further verification. Interestingly, obvious increases in mitochondrial protein *O*-GlcNAc modification induced by SalA-4g was significantly inhibited after 30 μM OSMI-1 intervention, with a decrease back to the basic expression level of the control group (Figure 7D). Together, these findings indicated that the increased level of mitochondrial *O*-GlcNAcylation can be safely inhibited by 30 μM OSMI-1 in neurons whose *O*-GlcNAc modification is activated, although its inhibitory effect on *O*-GlcNAcylation is not obvious under normal conditions.

We then examined the effect of O-GlcNAc-modified mitochondria induced by SalA-4g on mitochondrial stress response under OGD/R. An increase of mitochondrial ATP levels induced by SalA-4g in OGD/R neurons was completely abrogated by OSMI-1 (Figure 7E). Similarly, inhibition of mitochondrial *O*-GlcNAcylation levels blunted the protective effect of SalA-4g on mitochondrial oxidative stress and calcium surplus, respectively (Figure 7F,G).

**Figure 8:**
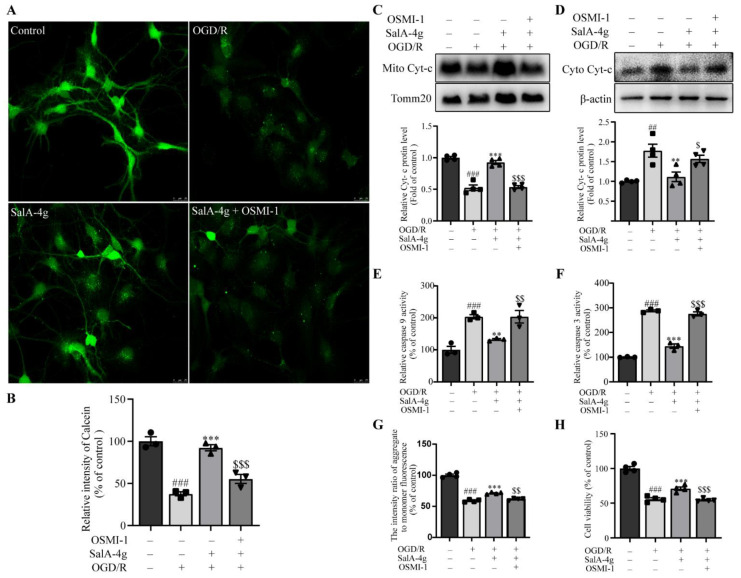
O-GlcNAcylation on mitochondrial proteins is involved in SalA-4g regulation of mitochondrial apoptosis pathway and neuronal survival in cortical neurons under OGD/R stress. (**A**,**B**) Representative fluorescence images (**A**) and quantification of average fluorescence intensity (**B**) of calcein in the control, OGD/R alone, OGD/R + SalA-4g, OGD/R + SalA-4g + OSMI-1, respectively. Scale bar, 25 μm. (**C**,**D**) Western blot analysis of cytochrome c expression in mitochondria (**C**) and cytosol (**D**) in cells after treatments, as mentioned above. (**E**,**F**) Histogram depicting the activation of caspase 9 (**E**) and caspase 3 (**F**) in different groups. (**G**) Quantitative analysis of the intensity ratio of JC-1 aggregate to JC-1 monomer fluorescence in different groups. (**H**) Histogram depicting the cell viability in different groups. All values are expressed as mean ± SEM (*n* = 3 or 4); ^##^ *p* < 0.01, ^###^ *p* < 0.001 versus control; ** *p* < 0.01, and *** *p* < 0.001 versus OGD/R alone; ^$^ *p* < 0.05, ^$$^ *p* < 0.01, and ^$$$^ *p* < 0.001 versus OGD/R + SalA-4g group.

Finally, we characterized the potential role of mitochondrial protein *O*-GlcNAcylation in SalA-4g neuroprotection through mitochondrial apoptosis pathway and neuronal survival. SalA-4g reduced the opening of MPTP in neurons after OGD/R insult, which was reversed by OSMI-1 (Figure 8A,B). Moreover, the inhibitory effects of SalA-4g on the cytochrome c release and caspase-cascade activation were also eliminated by OSMI-1 treatment (Figure 8C–F). Additionally, the recovery of MMP and cell viability induced by SalA-4g in OGD/R-injured neurons were all reversed by OSMI-1 (Figure 8G,H). Collectively, the above studies demonstrate that *O*-GlcNAc modification-mediated mitochondrial network homeostasis is a therapeutic target of SalA-4g neuroprotection.

## 3. Discussion

The present study conducted the first in vitro experiments to investigate role of *O*-GlcNAc-modified mitochondria-mediated mitochondrial network homeostasis in SalA-4g’s neuroprotection in neurons under ischemic-like conditions. To obtain a global overview of mitochondrial stress response under conditions of OGD/R, we conducted a comprehensive mitochondrial function screen, including mitochondrial structure, bioenergy, redox and calcium homeostasis, and mitochondrial pathway of apoptosis. Importantly, we explored roles of *O*-GlcNAc modification of mitochondrial proteins in modulating mitochondrial homeostasis and function under OGD/R stress in neurons, and identified mitochondrial energy metabolism pathway—*O*-GlcNAcylation as a key target for SalA-4g neuroprotection, which provides a new promising strategy for the treatment of ischemic brain injury.

*O*-GlcNAcylation, a nutrient-driven post-translational modifications in nucleoplasmic protein, has been newly identified as an endogenous prosurvival pathway, and thus acts as a promising target in ischemia/reperfusion injury, cardioprotection, and neuroprotection [7,28]. The recycling of *O*-GlcNAc modification is highly dependent on the HBP pathway, which is a branch of nutrient metabolism, including glucose, glutamine, and glucosamine. Therefore, therapeutic strategies that directly or indirectly elevate *O*-GlcNAc levels through HBP-associated *O*-GlcNAc signaling are being developed in experimental models of myocardial or cerebral ischemia, such as specific OGA inhibitor thiamet-G or glucosamine [29,30]. SalA-4g is a salidroside analogue synthesized in our laboratory by chemical structure-modification, with a phenyl ring containing a para-methoxy group and a sugar ring consisting of *N*-acetylglucosamine. A para-methoxy group on the phenyl ring increases molecular lipophilicity, which enables SalA-4g to penetrate BBB and exert stronger neuroprotective effects than salidroside [12]. Considering that *N*-acetylglucosamine is a substrate for HBP-associated *O*-GlcNAc cycling, we reasoned that regulation of *O*-GlcNAc modification is the key pharmacological target of SalA-4g neuroprotection. Indeed, our recent studies have shown that SalA-4g alleviates glucose fluctuation and elevates levels of protein *O*-GlcNAc, thus improving tolerance to OGD/R stress in cultured hippocampal neurons [14]. Here, we further focus on the regulation of mitochondrial *O*-GlcNAc modification induced by SalA-4g on mitochondrial homeostasis as a neuroprotective mechanism in neuronal cells under ischemic-like conditions.

Emerging evidence suggests that *O*-GlcNAc signaling contributes to mitochondria metabolism and homeostasis, as well as cellular stress response [31]. During myocardial ischemia-reperfusion injury, augmentation of *O*-GlcNAcylation alleviates mitochondrial dysfunction, including improving mitochondrial redox homeostasis, inhibiting MPTP formation and MMP loss, and maintaining mitochondrial bioenergy, thus contributing to cardioprotection [32,33,34]. Most recently, Park et al. report that mitochondrial protein *O*-GlcNAcylation is a critical process supporting functional extracellular mitochondrial secretion of astrocytes, which can promote neuronal survival and improve stroke outcome [35]. Firstly, we focus on evaluating the beneficial effects of SalA-4g on cellular bioenergy and mitochondrial network in neurons under OGD/R stress. As demonstrated by viability and apoptosis measurements, SalA-4g significantly increases neuronal viability and alleviates neuronal apoptosis, indicating an improvement of OGD/R stress tolerance in neurons (Figure 1A–D). Importantly, SalA-4g elevates total ATP content and mitochondrial ATP productivity in neurons under OGD/R stress (Figure 1E,F and Figure 2A,B). MitoTracker and TEM analysis further confirm the alleviated effect of SalA-4g on the loss of mitochondrial activity and structural integrity in neurons under OGD/R injury (Figure 2C,D and Figure 3).

Mitochondria play a prominent role not only due to their pivotal function in energy metabolism but also because specific pro-apoptosis proteins are located in the mitochondrion. When challenged by ischemic stress, oxidative stress caused by excessive ROS or antioxidant defense impairment can lead to lipid peroxidation and membrane permeability enhancement, resulting in cytosolic and mitochondrial Ca^2+^ overload. Mitochondrial oxidative stress and calcium paradox can induce MPTP opening and MMP depolarization, and finally activate the mitochondrial cytochrome c-caspase signaling pathway [36]. We therefore show here that SalA-4g can attenuate the mitochondrial dependent apoptosis pathway following OGD/R (Figure 4 and Figure 5).

In mouse models of forebrain ischemia/reperfusion or cardiac arrest/resuscitation, a transient elevation in *O*-GlcNAc modification can be seen in the cortex or hippocampus, suggesting the activation of an endogenous stress response pathway [8,37]. By comparison, the *O*-GlcNAc modification level of cortical neurons induced by OGD/R increases only slightly, indicating an impaired ability of neurons to activate this pro-survival pathway (Figure 6A,B). Importantly, SalA-4g elevates *O*-GlcNAc modification in neurons, both under normal and OGD/R stress, suggesting the beneficial role of SalA-4g in this stress response pathway.

In contrast to large number of studies on nucleocytoplasmic *O*-GlcNAcylation, the *O*-GlcNAc modification of mitochondrial proteins and its role in the regulation of mitochondrial function have just begun. An analysis of the *O*-GlcNAcome of cardiac mitochondria shows that mitochondrial proteins, especially those involved in oxidative phosphorylation system, are the major targets of *O*-GlcNAcylation [38]. In this study we therefore used mitochondria isolated from cortical neurons, to investigate the differential expression of *O*-GlcNAc levels on cells treated with or without SalA-4g. Our data reveal the upregulation of mitochondrial protein *O*-GlcNAcylation in neurons after SalA-4g treatment, both under normal and OGD/R stress (Figure 6C,D).

To verify the role of mitochondrial protein *O*-GlcNAcylation in SalA-4g-mediated mitochondrial network homeostasis under OGD/R conditions, OSMI-1, a selective inhibitor of OGT, is used to antagonize the upregulation of *O*-GlcNAcylation induced by SalA-4g. Since *O*-GlcNAc modification is necessary to maintain cellular processes [39], the inhibitory effect of 30 μM OSMI-1 on *O*-GlcNAcylation is proven to be effective and safe because the *O*-GlcNAcylation activation induced by SalA-4g has been completely inhibited, and notably, its expression maintains a basal level (Figure 7A–D). Finally, we further explore the impact of mitochondrial protein *O*-GlcNAcylation on mitochondrial network response under OGD/R stress. As expected, inhibition of *O*-GlcNAcylation with OSMI-1 can reverse the neuroprotective effects of SalA-4g, including mitochondrial network homeostasis and tolerance to stress (Figure 7E–G and Figure 8). These results suggest that the protective effect of SalA-4g is partly due to *O*-GlcNAcylation mediated mitochondrial network homeostasis. However, the characterization of *O*-GlcNAcylation on individual proteins in response to OGD/R stress and the identification of protein *O*-GlcNAcylation sites are still unknown. In future work, we will further explore the molecular mechanism of SalA-4g regulating *O*-GlcNAc modification under neuronal ischemic stress.

## 4. Materials and Methods

### 4.1. Drugs and Reagents

High purity SalA-4g (>98%) was prepared and characterized as described previously [40]. All reagents and materials provided by each kit are listed in each method.

### 4.2. Cell Culture and Treatment

Primary cortical neurons were prepared from Sprague-Dawley rat embryos at 16–18 days. Neurons were cultured in neurobasal medium supplemented with 2% B27 and 1× GlutaMAX in a humidified atmosphere perfused with 95% air and 5% CO_2_ at 37 °C. The whole culture process was maintained for 7–8 days, and the medium was renewed every 2–3 days [41].

The induction of OGD/R was based on the method as described elsewhere [42]. Mature cortical neurons were incubated with glucose-free DMEM, and placed in an anaerobic incubator (a gas mixture containing 1% O_2_ and 5 % CO_2_) for 2.5 h at 37 °C. Reoxygenation was performed by replacing OGD medium with neuronal culture medium and returning cultures to normoxic conditions for 24 h.

For SalA-4g treatment, different doses of SalA-4g were added at the initial stage of OGD, and maintained during the whole process of OGD/R. The control group was maintained in neuronal culture medium under normoxic atmosphere. To investigate the involvement of *O*-GlcNAcylation on the neuroprotective effects of SalA-4g, different concentrations of OSMI-1 (a cell permeable inhibitor of OGT, Sigma-Aldrich, St. Louis, MO, USA) were added to normal neurons or maintained in the process of OGD/R injury with or without SalA-4g.

### 4.3. Cell Viability Assay

The viability of primary cortical neuron after different treatments was evaluated by the CCK8 kit (Dojindo, Kumamoto, Japan). Neurons were incubated with 10% CCK8 reagent under normoxic conditions for 2.5 h. The formation of water-soluble orange formazan was detected by reading the absorbance at 450 nm using a microplate reader (Biotek, Winooski, VT, USA). Data were presented as the percent of the treatment group to control group.

### 4.4. LDH Release Assay

LDH leakage from cultured cortical neurons into the culture medium was determined by a commercial cytotoxicity assay kit (Beyotime, Shanghai, China). Briefly, 120 μL supernatant per well was collected and incubated with 60 μL detection reagent for 30 min at room temperature in the dark. The activity of LDH in the supernatant was determined by measuring the absorbance of formazan at 490 nm under a microplate reader (Biotek, Winooski, VT, USA). The group treated with 10% LDH releasing reagent was set as maximum LDH release. The LDH release was calculated as follows:

$$\%\mathrm{neuron}\;\mathrm{cytotoxicity}=\frac{\left[\mathrm{Sample}\;\left(\mathrm{OD}\right)\;-\;\mathrm{Blank}\;\left(\mathrm{OD}\right)\right]}{\left[\mathrm{Max}\;\left(\mathrm{OD}\right)\;-\;\mathrm{Blank}\;\left(\mathrm{OD}\right)\right]}\times 100\%.$$

Data were presented as the percent of the treatment group to control group.

### 4.5. MMP Assessment

MMP was measured using a JC-1 dye (Beyotime, Shanghai, China). Briefly, cortical neurons were incubated with 1 × JC-1 dye diluted in staining buffer for 20 min at 37 °C in the dark, followed by washing with ice-cold 1× staining buffer twice. JC-1 fluorescence intensity was observed with a TCS SP5 confocal microscope (Leica, Wetzlar, Germany). For accurate quantification, the red fluorescence intensity (JC-1 aggregates, excitation/emission = 525/590 nm) and green fluorescence intensity (JC-1 monomers, excitation/emission = 490/530 nm) were quantitatively analyzed with a microplate reader (Biotek, Winooski, VT, USA), and expressed as the ratio of red to green fluorescence.

### 4.6. Measurement of Cellular and Mitochondrial ATP Levels

The mitochondrial component of neurons was isolated with a mitochondria isolation kit (Thermo, Waltham, MA, USA). Intracellular total and mitochondrial ATP were evaluated using a luminescence-based ATP determination kit (Beyotime, Shanghai, China). Briefly, cortical neurons or mitochondria were collected and suspended in lysis buffer. After centrifugation, the supernatants of different groups were mixed with the ATP detection working solution. The intensity of luminescence was read in a microplate reader (Biotek, Winooski, VT, USA). The concentration of ATP in samples was measured according to the standard curve of ATP standard solution, and data were presented as percent of control.

### 4.7. Mito-Tracker Staining

The activity of mitochondria was detected by a red fluorescent dye MitoTracker (Thermo, Waltham, MA, USA) in live cells. Cultured neurons were loaded with 20 nM Mito-Tracker and 0.5 μg/mL Hoechst 33342 (Beyotime, Shanghai, China) for 30 min at 37 °C in the dark. Dye solution was discarded, and cultures were washed and immersed in pre-warmed fresh medium, and then photographed with a TCS SP5 confocal microscope (Leica, Wetzlar, Germany).

### 4.8. TEM

For ultrastructural observation of mitochondria, cell samples were processed for TEM assay according to routine procedures. Briefly, cortical neurons were collected and fixed in 2.5% glutaraldehyde in 0.1 M sodium cacodylate buffer for 2 h at 4 °C. Ultrathin osmium-stained sections were prepared, and then examined under a HT-7700 transmission electron microscope (Hitachi, Tokyo, Japan).

### 4.9. ROS Levels

Intracellular ROS generation was quantified using a fluorescent probe DCFH-DA (Sigma-Aldrich, St. Louis, MO, USA). Briefly, cells were reacted with 10 μM of DCFH-DA for 20 min at 37 °C in the dark, and then rinsed three times with HBSS. The fluorescence intensity of DCF (DCFH-oxidation) was quantitatively analyzed with a microplate reader (excitation/ emission = 488/525 nm).

### 4.10. Mitochondrial Superoxide Analysis

MitoSOX^TM^ Red reagent (Invitrogen, Carlsbad, CA, USA) was used to assess mitochondrial superoxide production in living neurons. Briefly, cortical neurons were loaded with the probe (5 μM) for 10 min in the dark, and then rinsed twice with HBSS buffer. The fluorescence intensity of MitoSOX was quantified by a microplate reader (excitation/emission = 510/580 nm).

### 4.11. Measurement of SOD, GSH, and GSSG

For SOD activity detection, cortical neurons were collected and suspended in lysis buffer provided by the total SOD assay kit (Beyotime, Shanghai, China). After centrifugation, the resulting cell supernatant was collected and mixed with WST-8/enzyme working solution. Total SOD activity was determined by calculating the absorbance at 450 nm. For measurement of GSH and GSSG, samples were processed according to the instructions of the GSH and GSSG assay kit (Beyotime, Shanghai, China), and the absorbance at 412 nm was measured under a microplate reader (Biotek, Winooski, VT, USA). Data were presented as the ratio of GSH to GSSG.

### 4.12. Measurement of Intracellular Ca^2+^ Level

Intracellular calcium levels were measured via a Fluo-4 AM probe (Thermo, Waltham, MA, USA). Cortical neurons were loaded with 5 μM Fluo-4 AM for 30 min at 37 °C. To ensure the complete transformation of Fluo-4 AM into Fluo-4, cultures were washed and maintained in culture medium for additional 30 min. Fluo-4 fluorescence was detected with a microplate reader (excitation/emission = 488/525 nm).

### 4.13. Measurement of Mitochondrial Ca^2+^ Concentration

Intramitochondrial calcium concentrations in cortical neurons were assessed by a mitochondria-selective Ca^2+^ indicator Rhod-2 AM (Thermo, Waltham, MA, USA). Briefly, cortical neurons were reacted with 4 μM Rhod-2 AM for 40 min at 37 °C, and then washed and incubated for a further 30 min to guarantee complete deesterification of intracellular AM esters. Rhod-2 fluorescence was detected in a microplate reader (excitation/emission = 550/580 nm).

### 4.14. Analysis of MPTP Opening

The MPTP opening in mitochondria was detected with a MPTP assay kit (Beyotime, Shanghai, China). For the indication of green fluorescence only in mitochondria, cortical neurons were rinsed with PBS and then loaded with calcein acetoxymethyl ester (calcein-AM) and calcein fluorescence quenching agent CoCl_2_ for 30 min in the dark. After loading the probe, neurons were incubated in the fresh culture medium at 37 °C for an additional 30 min to ensure that calcein-AM was fully hydrolyzed by esterase to generate green fluorescent calcein. Cells were then washed and immersed in the assay buffer for the detection of fluorescence using a TCS SP5 confocal microscope (excitation/emission = 494/517 nm).

### 4.15. Preparation of Protein Samples

The protein extraction reagent and a mitochondria isolation kit (Thermo, Waltham, MA, USA) were used for total protein extraction, and isolation of mitochondrial and cytoplasmic proteins, respectively. Protein concentration was estimated with a BCA protein assay kit (Thermo, Waltham, MA, USA), and samples were stored at −20 °C.

### 4.16. Western Blot

Protein samples were electrophoresed on 4–15% Tris-Gly gels and transferred to PVDF membranes (Millipore, Bedford, MA, USA). After 1.5 h blocking with 5% bovine serum albumin (*w*/*v*) or 5% (*w*/*v*) slim milk in TBS/T at room temperature, membranes were incubated overnight at 4 °C with primary antibodies, which were listed as follows: *O*-GlcNAc (CTD110.6) (1:1000, CST), Bax (1:1000, Abcam), Bcl-2 (1:1000, Abcam), Cytochrome c (1:1000, CST), Tomm20 (1:2000, Abcam) and β-actin (1:2000, Abcam). After washing, membranes were exposed to the corresponding secondary antibody (1:5000, Sigma-Aldrich, St. Louis, MO, USA) at room temperature for 1 h. Protein bands were detected under a 5200 multi-imaging system (Tanon, Beijing, China) with the ECL chemiluminescence reagents (Tanon, Beijing, China). Quantitative analyses for gray value of protein bands were performed with ImageJ software (NIH).

### 4.17. Caspase 3 and Caspase 9 Activity Assay

The activation of caspases was detected using colorimetric assay kits (BioVision, Palo Alto, CA, USA). Briefly, cortical neurons were harvested and lysed on ice for 15 min. After centrifugation, the resulting supernatants were collected as fresh 1.5 mL centrifuge tubes for protein concentration assay. Then, the labeled substrates DEVD-*p*NA and LEHD-*p*NA were used to assess the activity of caspase 3 and caspase 9, respectively. The absorbance of *p*NA catalyzed by caspases in the sample was measured at 405 nm using a microplate reader (Biotek, Winooski, VT, USA).

### 4.18. Statistical Analysis

Histograms were created by GraphPad Prism 8.3 and expressed as mean ± SEM. Statistical significance was assessed using one-way ANOVA followed by Tukey’s post hoc analyses. *p* < 0.05 was considered as statistically significant.

## 5. Conclusions

In summary, we systematically evaluated the role of SalA-4g contributing to the positive outcome in the regulation of stress-related cellular energy metabolism disorders and mitochondrial homeostasis imbalance in neuronal cells under ischemic-like conditions. A new strategy was proposed for the first time, stating that mitochondrial protein post-translational modification *O*-GlcNAcylation may be critical in regulating mitochondrial network structure homeostasis in hypoxia-ischemia induced neuronal damage. Further, boosting the level of mitochondrial protein *O*-GlcNAc by SalA-4g significantly improved mitochondrial energy homeostasis and inhibited mitochondria-dependent apoptosis, providing an in-depth insight into the pharmacological mechanism of SalA-4g for the treatment of cerebral ischemia.

## Data Availability

The data presented in this study are available on request from the corresponding author.

## Sample Availability

Samples of the compounds are available from the authors.
